# Overweight and Obesity in Children of Immigrant Versus Native Parents: Exploring a Local Setting in Portugal

**DOI:** 10.3390/ijerph17217897

**Published:** 2020-10-28

**Authors:** Susana Moreira, Luzia Gonçalves

**Affiliations:** 1 InHealth Consulting, Maputo 1100, Mozambique; susana.berjano.moreira@gmail.com; 2 Global Health and Tropical Medicine (GHTM), Instituto de Higiene e Medicina Tropical, Universidade Nova de Lisboa, 1349-008 Lisboa, Portugal; 3 Centro de Estatística e Aplicações da Universidade de Lisboa (CEAUL), 1749-016 Lisboa, Portugal

**Keywords:** children of immigrants, parents’ origin, overweight and obesity, risk of abdominal obesity

## Abstract

In Portugal, the majority of immigrants come from Portuguese-speaking countries. Among children, overweight and obesity are serious public health concerns, but a few studies include children with immigrant background. This study aimed to estimate the prevalence of overweight and obesity and risk of abdominal obesity in school-age children and to explore potential determinants, considering the origin of the children’s parents (both mother and father are non-Portuguese, only one Portuguese, and both are Portuguese). A cross-sectional study included a random sample of 789 children (5–11 years old, 49.2% of males and 50.8% female) attending public primary schools in Barreiro, district of Setubal, Portugal. Fieldwork occurred from 20th April to 3rd July 2009. Data concerning socioeconomic, eating habits, and sports outside school were obtained through a questionnaire applied to the children’s person in charge. Anthropometric measures were collected by certified technicians. One-third of the children presented overweight and obesity (33.7%, 95% CI [30.0; 37.7]) and risk of abdominal obesity (16.4%, 95% CI [13.6; 19.7]) without differences according to parents’ origin. Children with immigrant background presented higher consumption of low-priced, high-sugar, and high-fat foods, with a worse situation for girls. Females from both non-Portuguese parents also practiced less sports outside school than those where one or two parents are Portuguese. Thus, promoting and monitoring a healthy diet and physical activity in this group should be prioritized in this local setting.

## 1. Introduction

Overweight and obesity in children has been associated to several diseases in adult life, such as cardiovascular diseases, diabetes, hypertension, hypercholesterolemia [1,2,3,4]. In children, serious psychological and social consequences (e.g., lowered self-esteem and bulling in school) were also reported [5,6]. These consequences may be amplified with a serious impact on children when they simultaneously accumulate an immigration background and obesity.

Although the prevalence of overweight and obesity in children appears to be flattening in some European countries, in Portugal, some researchers argue that it is premature to consider that obesity in children is stabilizing [7,8]. The National Program for the Promotion of Healthy Eating highlighted a decreasing trend in prevalence of overweight and obesity, in children aged 6–8 years old, from 37.9% in 2008 to 29.6% in 2019 [9]. However, Portugal is still one of the European countries with the highest prevalence of overweight and obesity in children [8,10,11]. Although there are several local studies about overweight and obesity (e.g., in schools), few have explored overweight and obesity in immigrant children and its sociocultural influences and unhealthy behaviors [10]. Moreover, data collection in different time moments, their analysis, and interpretation are crucial to understand overweight and obesity trends in immigrant and native children. In 1993, 2009, and 2013, André et al., 2017 found a lower risk of overweight and obesity for Cape Verdean children compared with native children (aged 6–12 years) in a deprived neighborhood in Lisbon area.

The puzzle of immigration in Portugal is different from other European countries. Even though due to the financial crisis from 2008 to 2010 Portugal saw a change in the number and profile of immigrants and foreign residents, the majority of immigrants continues to come from the Community of Portuguese-speaking Countries (CPLP), particularly from Brazil and from the African countries of Portuguese official language [12].

Migration to Western societies seems to increase the risk of overweight and obesity, especially due to lifestyle alterations. Children that belong to communities with a high percentage of immigrants or with low socioeconomic status have a higher risk to develop obesity [13,14]. Studies have shown that obesity is more frequent in successive generations than in the first generation. Over time, immigrant populations assimilate certain attitudes and behaviors of the host country’s population [15,16]. Changing in diet patterns, that some immigrant populations are exposed, may in part explain the health status of these populations. Immigrants tend to engage in a more sedentary way of life, having lower access to the practice of sports, and they abandon their traditional food habits and adopt Westernized dietary patterns [13,17]. Immigrants tend to be poorer, with low wage jobs and unstable employment and consequently tend to access to low-priced, high-sugar, and high-fat foods [18,19,20].

Further research is needed to understand the role of parental socioeconomic factors in the prevalence of overweight and obesity in children with immigration background, in order to design better community-based interventions at the local level [21]. The lack of studies in Portugal about children of immigrants, mainly from low-and-middle-income countries and the geographical variations within the country, in terms of overweight and obesity, reinforce the need for regional and local studies [22,23]. In this work, we present data collected in 2009, in Barreiro—a municipality located south of Lisbon—including native and children with immigration backgrounds. Lisbon, Faro, and Setúbal presented the highest proportion of immigrants in Portugal. According to official data, in 2019, these three districts totalized 405,089 resident citizens (68.6% of the total) and Setúbal (where Barreiro is located) accounts to 51.983% (+29.3% compared with 2018) of immigrants [24,25]. School-age children of the public sector in Barreiro were evaluated in order to identify the association between nutritional status and a set of variables, including outdoors physical activity and food intakes, considering the origin of their parents. The last one has a central role in the main null hypotheses: (i) There no differences in terms of overweight and obesity and risk of abdominal obesity among three groups of children: “both mother and father are non-Portuguese,” “only one is Portuguese,” and “both are Portuguese,” (ii) children from immigrant origin and native children present similar anthropometric measures, and (iii) the migratory origin of parents does not influence some potential determinants of overweight and obesity in children of this particular setting.

## 2. Methods

### 2.1. Sample Size and Variables 

A cross-sectional study was conducted in the public primary schools, in Barreiro (about 3639 km^2^ and 78,764 inhabitants in 2011), Setúbal, Portugal. Data collection occurred from April 2009 to July 2009, through a questionnaire and anthropometric assessments.

Sample size calculations were based on an estimated prevalence of overweight and obesity around 0.53, from an unpublished study with children attending in the health center. This estimate is near to the 0.50, which provides the maximum sample size. According to official data of the last school year (2007/08), public primary schools accounted 3049 children. Thus, considering a confidence level of 95%, a desired precision of 3.7%, and 30% of non-response rate or other type of losses, the target sample size (*n* *) was estimated in 809 children. The type of variables included in the applied questionnaire, budget, time, and human resources were also considered in final sample size [26].

**Figure 1:**
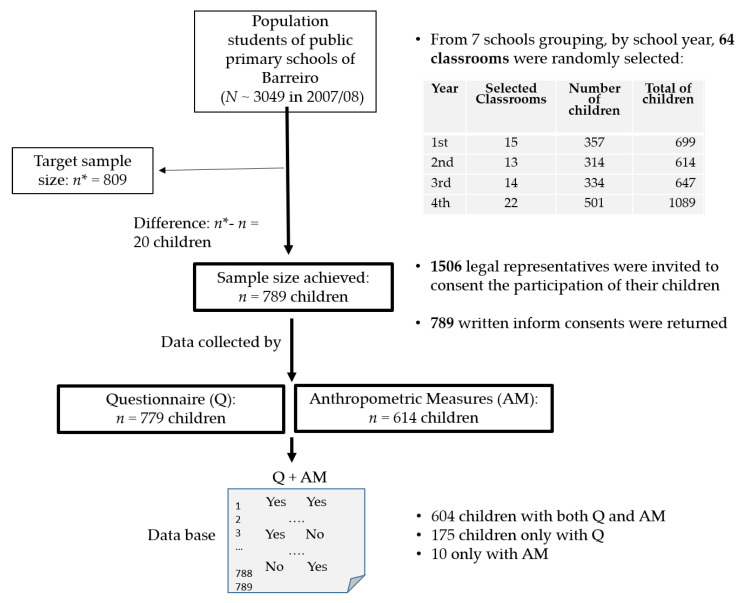
Sampling and data collection of this cross-sectional study.

Figure 1 describes some sampling and data collection steps, during the fieldwork. A total of 789 children (5–11 years old) participated in this study. Children whose parents or legal guardians did not return written informal consent were not able to participate in this research. The questionnaire was filled by 779 parents or legal guardian. In terms of the anthropometric measures, 614 children were assessed by certified technicians. The complete cases (questionnaire and anthropometric measures) were achieved to 604 children. For 175 children, we only obtained data from the questionnaire but the anthropometric measures were not performed. Yet, for 10 children were evaluated by technicians, but their questionnaires were not returned or were not considered valid. 

Socioeconomic data, physical activity outside school, and dietary habits were collected from a questionnaire filled by the children’s parents or legal guardians. Some food consumptions were addressed through “Which of the following item food does your child usually eat every day or almost every day?”. The food item list included soup, meat, fish, eggs, fruit, salad, bread, breakfast cereals, milk, yogurt/cheese, soft drinks, sweets, fries, and fast-food.

Anthropometric data (e.g., weight, height, skinfolds, and waist circumference) was collected by certified technicians, following the recommendation of the International Society for the Advanced of Kinanthropometry (ISAK), in order to minimize technical errors, according to standardized procedures [27]. The Centers for Disease Control (CDC) definition was used to establish cutoff points for body mass index (BMI) for age and sex to obtain the nutritional status [28,29]. For analytic purposes, nutritional status was grouped into three categories: underweight (<5th percentile), normal weight (5th–85th percentile), and overweight and obesity (>85th percentile). 

Waist-to-height ratio (WHtR) was recoded into a binary indicator to classify each child as having no risk (WHtR < 0.50) or having risk of abdominal obesity (WHtR ≥ 0.50) [2]. 

Regarding the children’s parents, based on which country they were born in rather in law and constitution, was recorded into: (i) both mother and father non-Portuguese, (ii) only one Portuguese, and (iii) both are Portuguese. This categorical variable was used to define roughly the “immigration background” of our children ((i) + (ii)) versus native children [(iii)]. For example, in the EU Statistics on Income and Living Conditions (EU-SILC) “children with an immigrant background are considered to be those who live in a household with at least one foreign-born adult” [30]. In our work, “adult” means only the father or mother because we only collected information on the country of birth for father, mother, and child, separately.

### 2.2. Statistical Analysis

Data analyses were made using SPSS 22.0 (IBM, Armonk, NY, USA). The descriptive analysis of qualitative variables was summarized by absolute (*n*) and relative (%) frequencies. Quantitative continuous variables (e.g., several anthropometric measures) were described using means and standard deviations (SD). In cases of an asymmetric distributions, the median and interquartile interval (P_25_–P_75_) were also presented. EpiTools program [31] was used to obtain 95% confidence intervals (95% CI) for the prevalence of overweight and obesity and abdominal obesity, using the Wilson method [32]. To compare anthropometric measures for two or three independent groups, Student’s *t*-test or one-way analysis of variance was performed, after testing the normality (Kolmogorov-Smirnov and Shapiro–Wilk tests) and homogeneity of variances (Levene test). In case of failure, Mann–Whitney–Wilcoxon (MWW) and Kruskal–Wallis (KW) tests were used. Relations between nutritional status and several categorical variables were analyzed through chi-square tests. A *p*-Value (*p* < 0.05) was considered statistically significant for all tests.

In addition, adjusted odds ratio (OR_a_) were obtained by multiple logistic regression models to address confounding. Hosmer and Lemeshow test and Cox and Snell R^2^ and Nagelkerke R^2^ measures were calculated.

### 2.3. Ethical Approval

Ethical aspects were taken into account in this research. Authorizations from the health deputy and the school representatives were ensured to collect the questionnaire applied to parents or legal guardians. The children’s representatives signed the written consent for the participation in this study. This research was supervised by the Scientific Council of the Institute of Hygiene and Tropical Medicine because the institutional ethical committee was established in 2010, after the data collection. 

## 3. Results

### 3.1. Sociodemographic Characteristics and Prevalence of Overweight and Obesity in Children, Considering the Parents’ Origin 

The total sample included 789 children. Table 1 shows the country of birth of the children and their parents. Angola and Cape Verde were the most representative countries for fathers and mothers of our children. Regarding parents’ country, 19.8% of the mothers and 20.2% of the fathers were born outside Portugal. Only 5.6% of the children were born in a foreign country. 

Table 2 presents some sociodemographic characteristics. Briefly, 49.2% were male and 50.8% female, aged between 5 and 11 years (mean ± SD: 7.99 ± 1.55). In terms of siblings, 24.3% are only child, 51.5% have one brother or sister, and 9.9% have at least three brothers or sisters (maximum = 6). 

Regarding household members (*n* = 697), 5.7% (*n* = 40) of children lived with one adult, 78.3% (*n* = 546) lived with 2 or 3 people, and 15.9% (*n* = 111) lived at least with 4 people (data not shown). 

Combining the origin of the child’s parents, for 12.4% (*n* = 92) of the children, their mother and father are non-Portuguese; for 15.6% (*n* = 115), only one is Portuguese, and for 72.0% (*n* = 531), both parents are Portuguese. Then, according to our definition, children with immigration background represent 28.0%. The frequency of single-siblings is similar in those three groups (χ^2^ = 4.472; *p* = 0.11). However, significant differences were found for median household sizes in the three groups (KW: *p* < 0.001), with the first group (children with mother and father non-Portuguese) presenting a higher household size, but for a similar number of rooms in their houses (KW: *p* = 0.46, data not shown). 

Table 3 outlines the nutritional status by sex, age, number of siblings, and origins of the child’s parents. Similar percentages of overweight and obesity were found in the categories of each variable. Adding an estimate by interval, the overall prevalence of overweight and obesity was 33.7%, 95% CI [30.0; 37.7].

**Figure 2:**
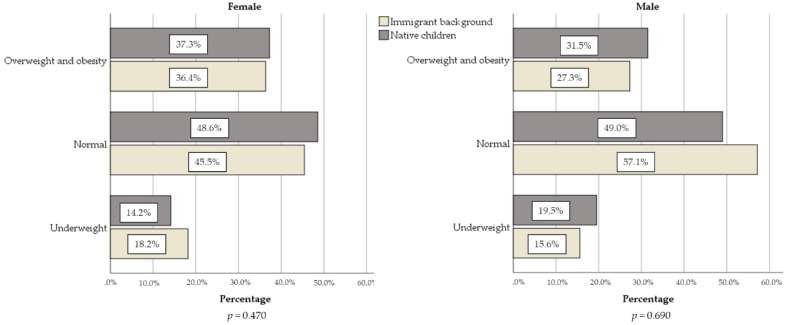
Nutritional status, according to CDC definition, for children with immigration background or native, separated by sex.

Considering the BMI-for-age percentiles by sex, no differences were found in nutritional status for native children and children with immigration background (mother or father non-Portuguese or only one Portuguese) in females (*p* = 0.470) and males (*p* = 0.690). The prevalence of underweight was almost half the values for overweight and obesity in native and also for children with immigration background. This pattern highlights in Figure 2.

### 3.2. Anthropometric Measures and Abdominal Obesity Risk 

Although, in terms of the nutritional status of children, no significant differences were found in males and females according to parent’s origin, it is important to detail other anthropometric measures. Table 4 presents several measures for children with immigration background ((i) + (ii)) and native children ((iii)) separated by sex, as mentioned in Methods section. This option by two groups (instead of three) allows facilitating the presentation of the findings and to avoid small sample sizes. 

For males, Table 4 shows similar mean values in almost all measures in the children with immigration background and natives. For females, some differences were found (at 5% level), in terms of tricipital (by T-test: *p* = 0.03, but not by MMW test: *p* = 0.07), thigh skinfolds (*p* = 0.04), and WHtR (*p* = 0.04), with lower mean values for children with immigration background. Given the last result, it is important to detail the abdominal obesity indicator based on WHtR. For females, 12 (15.4%) in the children with immigration background and 42 (19.9%) in the native group presented risk of abdominal obesity, without significant differences (*p* = 0.38). Similar to males, with 9 (11.7%) and 30 (15.0%) for children with immigration background and native groups, respectively (*p* = 0.48). Within each group, comparing males and females, significant differences were not found. Thus, findings suggest an overall estimate for abdominal obesity risk of 16.4% with 95%CI [13.6, 19.7].

### 3.3. The Role of the Parent’s Origin in Some Potential Direct or Indirect Determinants of Obesity in Females and Male

Despite this similar situation between groups, parent’s origin seems to be an important variable due to its relationship with some variables that may be potential direct or indirect determinants of obesity. Table 5 presents the physical activity outside school, housekeeping tasks, and some food consumption among the three groups of children for total sample and stratified by sex. Regarding physical activity outside school, a gradient was found. If both parents are non-Portuguese, 33.0% of the children practice sports outside the school. If only one parent is Portuguese, 41.7% of the children practice it, and if both parents are Portuguese, 50.9% of the children practice it (χ^2^ = 11.32; *p* < 0.01). This gradient is strongly marked for female. Female children with both parents or only one with non-Portuguese nationality practice less sport outside school than the daughters of both Portuguese parents (χ^2^ = 15.31; *p* = 0.001). For boys, frequencies of practice sports outside the school were similar among groups. Children with both parents non-Portuguese help more in daily housekeeping tasks, 87% (*n* = 67), than those whose parents are both Portuguese, 71.2% (*n* = 306), or when only one is Portuguese, 68.8% (*n* = 66). The relation between doing housekeeping and parents’ nationality is statistically significant (χ^2^ = 9.23; *p* = 0.001) for the total sample. 

Table 5 also outlines the link between food intakes (every day or almost every day) and parents’ origin. If both parents are non-Portuguese, they reported higher percentages of the children that consume low-priced products, namely, eggs, fries, fast-food, sweets, and soft drinks. For male children, contrary to females, no differences were found among groups, in terms of sweets and fast-food consumptions at 5% level. Particularly, for females from both parents non-Portuguese, more than 70% reported consumptions of high-sugar products (soft drinks: 72.1% and sweets: 75.0%) every day or almost every day. 

**Figure 3:**
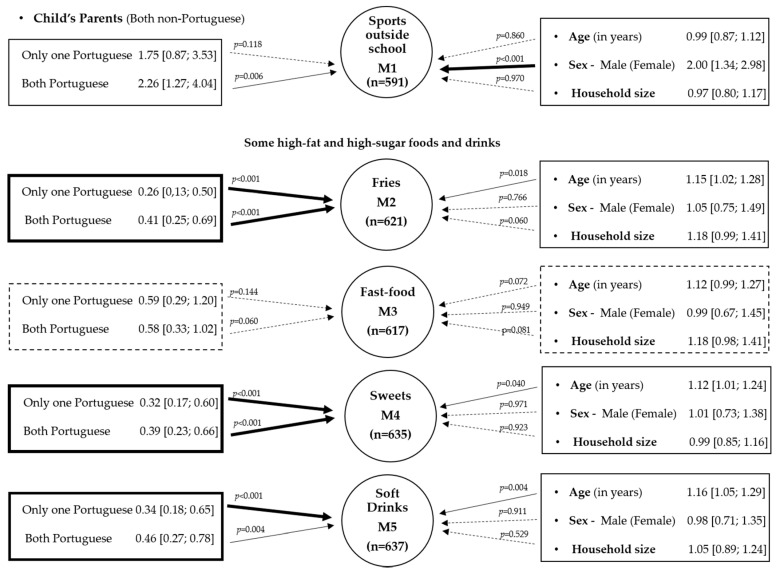
Results of five models (M1–M5). Each dependent variable is within circles and explanatory variables (or their categories) are within rectangles. Values correspond to adjusted odds ratio (with 95% CI). Thick line arrows indicate very significant associations (*p* < 0.001), solid line arrows show significant associations (with 0.001 < *p* ≤ 0.05), and dashed lines denote no association (*p* > 0.05).

Figure 3 highlights the role of the origin of the child’s parents in the practice sports outside school and some high-fat and high-sugar consumptions (fries, fast food, sweets, and soft drinks), adjusting for age, sex, and household size, using five models. 

In all models, we considered “Both father and mother of the child are non-Portuguese” as the reference category. From model M1, the adjusted odds ratio indicated that children with father and mother of the Portuguese origin, compared with non-Portuguese parents, had 2.26 times more chance to practice sport outside school. On the right side, Figure 3, a significant association with sex revels that the odds of practicing sport outside school for male children is 2.0 times that of the female children. Models M2, M4, and M5 revealed a disadvantageous situation for children with non-Portuguese parents in terms of fries, sweets, and soft drinks consumptions (every day or almost every day). In addition, these models revealed associations with age. According to the fitted models, for a 1-year increment in age, the odds of fries, sweets, and soft drinks consumptions seem to be increased by 15%, 12%, and 16%, respectively. Although no significant associations were obtained at 5% level, household size seems also to reveal some influence in sweets and fast-food consumption (*p* < 0.10). Association between parents’ origin and fast-food was not significant (at 5%), but there is a similar trend to the previous models, showing a protective situation for children when both parents are Portuguese in opposite to non-Portuguese parents.

Among low-priced products, the consumption of the eggs was omitted in Figure 2 due to its nutritional value, but other models also revealed an association with parents’ origins (*p* < 0.01) and with household size (ORa = 1.21; *p* = 0.03; 95% CI: [1.02; 1.43]). Analyzing some food and drinks intakes and their nutritional status, only soft drinks seem to be associated with overweight and obesity. Exploring this relationship by sex, only a significant association was found for females (χ^2^ = 14.31; *p* = 0.001). Multiple logistic regression models were also explored to explain the presence of overweight and obesity through a set of potential explanatory variables, and only a statistical association between soft drinks and overweight and obesity was found (ORa = 1.67; *p* = 0.02; 95% CI: [1.05; 2.66]).

## 4. Discussion

In the municipality of Barreiro, with immigrants mainly from low- and-middle-income countries of CPLP, no differences were found in terms of overweight and obesity and abdominal obesity risk in children of public schools, according to parents’ nationality. However, parents’ nationality seems to play a crucial role in some potential protective and risk factor for obesity. By sex, it seems to be a tendency for the females to have more overweight and obesity than males, however, this difference was not significant. The same occurred for abdominal obesity risk. 

Contrary to immigrant children, we found some studies about overweight and obesity and its determinants for Portuguese children of slightly different ages [28,33,34,35,36,37]. For male children (6–10 years old), the prevalence of overweight varied from 14.7% to 30.5% and obesity from 5.3% to 13.2%. In girls, overweight varied from 16.5% to 29.1% and obesity from 6.4% to 12.6% [28]. In addition to Portuguese children (6–8 years old), overweight prevalence was 32.2–95% CI [29.6; 34.9] and obesity was 14.4–95% CI [11.7; 17.6], following CDC definition [8,11]. Among 7–9 years old school children in Aveiro, Pedrosa et al. [28] reported prevalence of excess weight between 28% and 31% according to International Obesity Task Force (IOTF) and CDC definitions, respectively. Gomes et al. [33] summarized studies between 2004 and 2013 and a moderate-to-high prevalence values in 9–11 years old were reported in national samples (*n* ranging from 405 to 3584). 

In Portuguese school-age children, findings about abdominal obesity and detailed anthropometric measures among children with immigrant background (and natives) are rare. In 2013–2014, Rodrigues et al. [38] found a prevalence of 21.9% and Albuquerque et al. [39] found 23.6% in central region of Portugal. Both studies presented higher values compared with our study. André et al. [10] presented height, sitting height, weight, BMI, and skinfolds for children in a deprived neighborhood in 1993, 2009, and 2013, concluding that Cape Verdean children had a lower risk of being overweight or obese than native children. Our children with an immigrant origin, on average, were younger and seem to present higher mean values of the mentioned measures for males and females. This advantageous situation was not found here and almost all anthropometric measures were similar for children with immigrant background and native group. About a one-third of our children (5–11 years old) presented overweight and obesity, without significant differences, according to parents’ nationality. Data also reveal difference between “underweight” and “overweight or obesity” (Figure 2). The prevalence value for overweight or obesity is almost twice of underweight, even in children with an immigrant background. For Portuguese children (5–13 years old), an even more discrepant pattern has already been described [40] and also in studies in low and middle-income countries [41]. 

Angola was one of the most frequent countries of origin (Table 1). This may bring a hidden and interesting finding. Angola was devastated by a 40-year period of war until 2002, forcing migration flows and high levels of chronic malnutrition continue to be a serious health problem nowadays. Fathers and mothers of our study (data collected in 2009) probably lived this war period. Angola continues to point out a high proportion of children suffering from stunting (37.6%) and wasting (4.9%), particularly in under-five children [42]. Our children with parents from African countries seem to move to another extreme in terms of nutritional status, suffering also from overweight and obesity as the native children (aged 5–11 years).

Fitted models highlighted the importance of parents’ nationality direct or indirectly through several variables such as the practice of sports outside school, fries, sweets, and soft drink consumption, which may eventually, in middle and long terms, reflect in the children’s nutritional status. Of course, fitted models were poor, which is likely due to the complexity and multifactorial nature of the food consumption. However, age was associated to an increase in fries, sweets, and soft drink consumption. Our findings need further consideration as high-sugar products consumption and less sports outside school were higher in female children compared with male children. Comparison of childhood obesity among groups brings different complexities regarding genetic, physiological, cultural, socioeconomic, and environmental issues, and the interaction between these variables is not completely understood [34].

Regarding physical activity, in Europe, immigrants and sons of immigrants have less tendency to be physically active and report a lower health status than nonimmigrants [15,43]. Among several possibilities, immigrant parents may face structural challenges (poor neighborhoods, busy schedules, language barriers) that makes difficult to have opportunities for physical activity [35,44]. Language is not expected to be a main barrier, because Portuguese is the main language in the Community of Portuguese-speaking countries. However, some local languages continue to be used by immigrants. 

Portugal is among one of the countries where the Mediterranean diet-like pattern fits, which is characterized by a protective effect in health. Nevertheless, studies should be undertaken to better understand the adoption of this diet by immigrant communities, where the links with the country of origin through their families and culture may result in different findings. 

Associations between childhood obesity and environmental characteristics highlight the importance of environmental determinants in urban areas and its relevance when delineating local and community interventions strategies in order to prevent childhood obesity [36]. Although the complexity of the determinants of obesity point to several directions, with underlying causes such as social and cultural aspects [13], in our study, it is highlighted that in the same condition, girls practice less sport outside the school and help slightly more their parents doing housekeeping than boys (in what regards to the parents’ origin). Still, little is known about gender-specific practices among immigrant parents and their children in Portugal. Some studies, in other settings, refer as plausible that immigrant parents may treat sons differently than daughters in leading to differences in the prevalence of overweight and obesity. Daughters may be more restricted than sons because of additional family obligations [16,45] and also by religious practices in some communities. Other studies alert for the social valorization of the overweight in African populations [46]. In particular, for women, this may promote unhealthy eating behaviors.

Regarding eating practices, studies refer that the overall diet in the Portuguese children tends to be low in carbohydrates and high in sugars and fat [35,36,37]. Dietary patterns that included eggs being reported to be consumed 1 or 2 times per week and fast food consumed occasionally or never were described by Rodrigues et al. [47]. In other study, Rodrigues et al. [48] found a lifestyle pattern that included sugar-sweetened beverages intakes (>2 times/week) was associated with the increased BMI and waist circumference. A recent work refers that in 7 out of the 24 countries analyzed, immigrant children are more likely to report very high levels of unhealthy eating (e.g., consumption of sweets and sugary drinks), with significant differences ranging from the low of 4.5 percentage points in Spain to the high of 8.7 points in Austria [30]. Millar et al. [49] studied immigrants from Sub-Saharan Africa in Australia and pointed out the replacement of traditional foods and an increased consumption of processed food and low ingestion of fiber. The same process is described in south Asians living in Europe [50]. Migration may, therefore, entail other nutritional habits [49,50]. Other studies suggest that even the pattern and speed of change in diet may differ from country to country and that immigrants belonging to the same ethnic group may be at different levels in the acculturation process [51,52,53,54]. In our study, even though an extensive analysis of the children’s diet was not undertaken, reported data on consumption of eggs, fries, fast food, sweets, and soft drinks is higher form children with immigrant background. Age was also associated to fries, sweets, and soft-drinks intakes. The association between nutritional status and soft drink consumptions was particularly important for female. Silva et al. [55] studied the effectiveness of the program to increase fruit/vegetable consumption and physical activity only in Portuguese children. Our study has a clear added value because it attempts to identify some aspects of immigrants’ health issues emphasizing the problem of overweight and obesity and abdominal obesity in children of immigrants in this municipality that may have an expression due to social and cultural background of their parents, adding to a growing body of literature that support local interventions focused on sex and culture specificity of immigrants. An open problem is also some variations with age. For example, considering children of immigrant mothers from low- and-middle-income countries, Zulfiqar et al. found sex-specific variations in prevalence with age, increasing more consistently for male than for female children [56].

Some limitations and strengths should be referred. BMI cutoff points is a central issue requiring careful comparisons and interpretations. In addition, the definition of immigration status varies from study to study [30]. An advantage of this study is related with measurements that were not self-reported but performed by technicians with ISAK certification, minimizing measurement errors [27,57]. However, eating habits, and practicing sports, among other, were self-reported by children primary caregiver and social desirability bias may be present. Data from 2009, may be a historical point to understand trends and processes at local level and contribute to inform researchers, policy makers, and public health practitioners about the evolution of prevalence of overweight and obesity. Additionally, it can be a baseline to future studies with children with immigration background to understand the nutrition transition at the community, household and individual level. Moreover, these children will be mothers and fathers in a near future. The impact of the ongoing introduction of several policies and measures in Portugal since 2010 also benefits from data collected before the implemented measures. For example, additional taxes on sugar drinks, a gradual decrease in the salt in bread, and prohibition on advertisement all food products, including beverages, with high energy values, salt, sugar, fatty acids, and processed fatty acids, to minors of 16 years old, is yet to be documented. Currently, most European countries, including Portugal, do not collect data systematically about the health status of immigrants. Better knowledge of the health of immigrants is essential to a country’s integration and health policies, but it is challenged by the lack of available data. Thus, at a local level, the study of successive generations can be useful to understand the acculturation process and risk factors in this particular setting or similar settings in order to offer guidance to health organizations to address the burden of obesity, its future comorbidities, and also monitoring some modified risk factors. 

## 5. Conclusions

In conclusion, approximately one-third of the children from public primary schools in Barreiro presented overweight and obesity. Abdominal obesity risk indicator also revealed the need for monitoring these children. In this study, no differences in terms of overweight and obesity were found in children according to parents’ origin. Age was associated with fries, sweets, and soft-drinks intakes. Particularly, for females from both parents non-Portuguese, more than 70% reported consumptions of high-sugar products every day or almost every day. Moreover, females from both non-Portuguese parents also practiced less sports outside of school. Thus, female children with both parents non-Portuguese seem to be a potentially vulnerable group in this setting. While Barreiro belongs to the Portuguese Healthy Cities Network based on the essential principles of the Healthy Cities Project, from the World Health Organization, which are Equity, Health Promotion, Community Participation, Intersectorial Cooperation, these local findings suggest that efforts should be made to tailor and monitor the municipality’s immigrant community, considering their specific characteristics.

## Figures and Tables

**Table 1:** Country of birth of the children and their parents.

	Child		Father		Mother	
**Country of birth**	***n***	**(%)**	***n***	**(%)**	***n***	**(%)**
Portugal	707	94.4	587	79.4	596	80.2
Angola	2	0.3	47	6.4	42	5,7
Cape-Verde	12	1.6	38	5.1	32	4.3
Guinea-Bissau	8	1.1	17	2.3	18	2.4
Mozambique	0	0.0	18	2.4	12	1.6
Brazil	10	1.3	13	1.8	14	1.9
São Tomé	2	0.3	4	0.5	4	0.5
Other countries	8	1.0	15	2.1	25	3.4

**Table 2:** Sociodemographic characteristics of children and origins of their parents.

Variables/Categories	Frequencies	
	*n*	%
**Sex**		
Male	388	49.2
Female	401	50.8
**Total**	**789**	**100.0**
**Age (in years)**		
5	38	5.0
6	125	16.5
7	121	16.0
8	157	20.7
9	185	24.4
10	104	13.7
11	27	3.6
**Total**	**757**	**100.0**
**Number of siblings**		
0	179	24.3
1	380	51.5
2	106	14.8
>=3	73	9.9
**Total**	**738**	**100.0**
**Child’s parents**		
Both non-Portuguese	92	12.5
Only one Portuguese	115	15.6
Both Portuguese	531	72.0
**Total**	**738**	**100.0**

**Table 3:** Nutritional status by subgroups.

	Nutritional Status				
Variables	Underweight	Normal Weight	Overweight and Obesity	Total	
	***n* (%)**	***n* (%)**	***n* (%)**	***n***	***p***
**Sex**					0.125
Male	55 (12.2)	157 (52.0)	90 (29.8)	302	
Female	48 (15.4)	147 (47.1)	117 (37.5)	312	
**Total**	**103 (16.8)**	**304 (49.5)**	**207 (33.7)**	**614**	
**Age (in years)**					0.093
5	5 (17.9)	17 (60.7)	6 (21.4)	28	
6	17 (17.0)	47 (47.0)	36 (36.0)	100	
7	11 (10.9)	61 (60.4)	29 (28.7)	101	
8	34 (25.2)	59 (43.7)	42 (31.1)	135	
9	17 (12.2)	68 (48.9)	54 (38.8)	139	
10	11 (17.5)	34 (54.0)	18 (28.6)	63	
11	3 (18.8)	6 (37.5)	7 (43.8)	16	
**Total**	**98 (16.8)**	**292 (50.2)**	**192 (33.0)**	**582**	
**Number of siblings**					0.261
0	16 (11.2)	74 (51.7)	53 (37.1)	143	
1	55 (18.6)	142 (48.1)	98 (33.2)	295	
2	14 (17.7)	46 (58.2)	19 (24.1)	79	
≥3	10 (19.2)	24 (46.2)	18 (34.6)	52	
**Total**	**95 (16.7)**	**286 (50.3)**	**188 (33.0)**	**569**	
**Child’s parents**					0.978
Both non-Portuguese	11 (17.2)	32 (50.0)	21 (32.8)	64	
Only one Portuguese	15 (16.7)	47 (52.2)	28 (31.1)	90	
Both Portuguese	69 (16.7)	201 (48.8)	142 (34.5)	412	
**Total**	**95 (16.8)**	**280 (49.5)**	**191 (33.7)**	**566**	

**Table 4:** Comparisons of variables (mean ± SD) between children with immigration background versus native parents, stratified by sex.

		Female			Male	
Variables (units)	Immigrant Background (*n* = 78)	Native (*n* = 212)	*p*	Immigrant Background (*n* = 77)	Native (*n* = 200)	*p*
Decimal age (years)	8.61 ± 1.49	8.81 ± 1.34	0.303	8.80 ± 1.49	8.68 ± 1.46	0.550
Weight (kg)	31.90 ± 9.03	32.07 ± 9.15	0.893	32.20 ± 11.18	31.29 ± 9.56	0.501
Height (cm)	133.88 ± 11.02	133.35 ± 9.59	0.689	134.02 ± 10.68	132.97 ± 10.25	0.449
Sitting Height (cm)	70.38 ± 4.90	70.80 ± 4.54	0.491	70.79 ± 4.63	70.92 ± 4.68	0.841
BMI (kg/cm^2^)	17.55 ± 3.48	17.77 ± 3.51	0.632	17.53 ± 3.94	17.36 ± 3.32	0.708
Body fat (%)	20.30 ± 8.30	22.57 ± 9.51	0.063	18.59 ± 10.29	18.33 ± 8.81	0.834
**Skinfolds**						
Tricipital	12.99 ± 5.29	14.56 ± 6.22	**0.032** ^**a**^	11.88 ± 6.50	11.58 ± 5.72	0.701
Thigh (mm)	20.19 ± 8.22	22.61 ± 9.02	**0.039**	17.81 ± 8.89	18.14 ± 8.87	0.785
Calf (mm)	13.71 ± 6.01	15.10 ± 6.57	0.104	12.21 ± 7.13	12.14 ± 6.55	0.930
Subscapular (mm)	9.56 ± 5.77	10.55 ± 6.83	0.258	9.10 ± 6.82	8.32 ± 5.90	0.343
Suprailliac (mm)	9.95 ± 6.23	11.47 ± 7.27	0.101	9.37 ± 7.91	8.59 ± 7.18	0.429
**Circumferences**						
Relaxed arm	20.64 ± 3.30	21.42 ± 3.66	0.100	20.44 ± 4.11	20.39 ± 3.93	0.925
Tensed arm	21.33 ± 3.19	21.98 ± 3.45	0.153	21.36 ± 3.89	21.24 ± 3.56	0.807
Calf (cm)	28.25 ± 3.75	28.74 ± 3.76	0.322	28.35 ± 4.05	28.15 ± 5.17	0.760
Waist (cm)	58.69 ± 7.20	60.41 ± 8.10	0.099	60.38 ± 9.19	60.37 ± 7.93	0.992
Hip (cm)	71.87 ± 9.21	72.55 ± 9.43	0.588	71.24 ± 10.72	70.36 ± 10.00	0.520
**Biepicondylar Breadths**						
Humerus (cm)	5.17 ± 0.46	5.19 ± 0.47	0.854	5.36 ± 0.55	5.29 ± 0.50	0.355
Femur (cm)	7.75 ± 0.66	7.71 ± 0.64	0.710	8.1 6 ± 0.79	8.08 ± 0.69	0.415
**Other measures**						
Waist-to-height ratio	0.45 ± 0.05	0.45 ± 0.04	**0.035**	0.45 ± 0.05	0.45 ± 0.04	0.568
Waist-to-hip ratio	0.82 ± 0.05	0.84 ± 0.07	0.062	0.85 ± 0.05	>0.87 ± 0.14	0.311
Arm muscle area	218.46 ± 53.56	226.34 ± 57.42	0.293	224.03 ± 67.59	225.40 ± 72.38	0.885
Arm fat area	124.44 ± 66.94	144.32 ± 80.17	0.071 ^b^	117.15 ± 88.26	113.09 ± 76.06	0.704

^a^ MWW test: median (P_25_–P_75_)—immigrant background: 12.50 (9.38–16.13) versus native: 13.00 (10.00–19.00); *p* = 0.073. ^b^ MWW test: median (P_25_–P_75_)—immigrant background: 111.85 (75.70–163.05) versus native: 122.80 (83.00–192.60); *p* = 0.071.

**Table 5:** Practice sports, housekeeping tasks, and food consumption according to parents’ origin, for total sample and stratified by sex.

Variables	Total and by Sex (*n*)	Percentage of Affirmative Responses			χ2 (*p*)
		Both Non-Portuguese (*n* = 92)	Only One Portuguese (*n* = 115)	Both Portuguese (*n* = 531)	
**Practice sports outside school**	**Total (716)**	**33.0**	**41.7**	**50.9**	**11.32 (0.003)**
	Female (364)	15.2	28.8	43.2	15.31 (0.001)
	Male (352)	52.4	55.7	58.7	0.69 (0.707)
**Housekeeping tasks**	**Total (603)**	**87.0**	**68.8**	**71.2**	**9.23 (0.010)**
	Female (323)	89.5	74.1	73.6	4.53 (0.104)
	Male (280)	84.6	61.9	68.3	5.48 (0.064)
Eggs consumption *	**Total (661)**	**65.1**	**38.7**	**39.0**	**20.23 (<0.001)**
	Female (333)	70.0	38.2	40.3	13.03 (0.010)
	Male (328)	60.5	39.2	37.6	7.92 (0.019)
French fries *	**Total (664)**	**58.0**	**23.6**	**33.5**	**25.41 (<0.001)**
	Female (336)	64.1	31.1	33.9	18.68 (<0.001)
	Male (328)	52.4	22.0	36.0	9.15 (0.001)
Fast food *	**Total (659)**	**38.2**	**22.0**	**22.1**	**9.48 (0.009)**
	Female (334)	42.1	23.6	19.9	9.12 (0.010)
	Male (325)	34.2	20.4	24.4	2.32 (0.313)
Sweets *	**Total (680)**	**67.9**	**39.6**	**43.4**	**18.71 (<0.001)**
	Female (349)	75.0	41.1	41.5	16.12 (<0.001)
	Male (331)	61.0	38.0	45.4	5.01 (0.082)
Soft drinks *	**Total (685)**	**72.1**	**44.9**	**51.4**	**15.92 (<0.001)**
	Female (348)	72.1	51.8	49.4	7.59 (0.022)
	Male (337)	72.1	37.3	53.5	11.38 (0.003)

* every day or almost every day.

## Abbreviations

CPLP	Community of Portuguese-speaking countries
ISAK	International Society for the Advanced of Kinanthropometry.
CDC	Centers for Disease Control
BMI	Body mass index
WHtR	Waist-to-height ratio
EU-SILC	EU Statistics on Income and Living Conditions
SPSS 22.0	Statistical Package for the Social Sciences version 22.0
CI	Confidence interval
MWW	Mann–Whitney–Wilcoxon
KW	Kruskal–Wallis
ORa	Adjusted odds ratio
SD	Standard deviation
IOTF	International Obesity Task Force
